# Patient and process factors associated with opportunities for improvement in trauma care: a registry-based study

**DOI:** 10.1186/s13049-023-01157-y

**Published:** 2023-11-27

**Authors:** Hussein Albaaj, Jonatan Attergrim, Lovisa Strömmer, Olof Brattström, Martin Jacobsson, Gunilla Wihlke, Liselott Västerbo, Elias Joneborg, Martin Gerdin Wärnberg

**Affiliations:** 1 Department of Global Public Health, Karolinska Institutet, Solna, Sweden; 2 Perioperative Medicine and Intensive Care, Karolinska University Hospital, Solna, Sweden; 3 Division of Surgery, Department of Clinical Science, Intervention and Technology (CLINTEC), Karolinska Institutet, Stockholm, Sweden; 4 Department of Physiology and Pharmacology, Karolinska Institutet, Stockholm, Sweden; 5 Department of Biomedical Engineering and Health Systems, KTH Royal Institute of Technology, Huddinge, Sweden; 6 Trauma and Reparative Medicine, Karolinska University Hospital, Solna, Sweden

**Keywords:** Trauma, Opportunities for improvement, Trauma care, Acute surgery

## Abstract

**Background:**

Trauma is one of the leading causes of morbidity and mortality worldwide. Morbidity and mortality review of selected patient cases is used to improve the quality of trauma care by identifying opportunities for improvement (OFI). The aim of this study was to assess how patient and process factors are associated with OFI in trauma care.

**Methods:**

We conducted a registry-based study using all patients between 2017 and 2021 from the Karolinska University Hospital who had been reviewed regarding the presence of OFI as defined by a morbidity and mortality conference. We used bi- and multivariable logistic regression to assess the associations between the following patient and process factors and OFI: age, sex, respiratory rate, systolic blood pressure, Glasgow Coma Scale (GCS), Injury Severity Score (ISS), survival at 30 days, highest hospital care level, arrival on working hours, arrival on weekends, intubation status and time to first computed tomography (CT).

**Results:**

OFI was identified in 300 (5.8%) out of 5182 patients. Age, missing Glasgow Coma Scale, time to first CT, highest hospital care level and ISS were statistically significantly associated with OFI.

**Conclusion:**

Several patient and process factors were found to be associated with OFI, indicating that patients with moderate to severe trauma and those with delays to first CT are at the highest odds of OFI.

## Introduction

Trauma is one of the leading causes of morbidity and mortality in all age groups globally [1, 2]. There are approximately 4.5 million global deaths each year due to trauma, [3] and it is one of the top contributors to disease burden worldwide, measured by disability-adjusted life years [4, 5]. Trauma is also resource-intensive as it is one of the most common reasons for critical care unit admission [6].

The quality of trauma care can be expressed using Donabedian’s quality of care framework, in which improved structures and clinical processes improve patient outcomes [7]. There are several methods to improve the quality of trauma care, with multidisciplinary morbidity and mortality review being a key method to address all components of this framework [8].

The morbidity and mortality review aims to assess the preventability of patient deaths and to identify opportunities for improvement (OFI) in the structure and clinical processes of trauma care. The rate of OFI in trauma deaths ranges between 20 and 76%, [9–11] and common OFI relates to airway management, management of traumatic brain injury, fluid resuscitation, delays in prehospital transport and delays to surgery [12–15].

Whereas numerous studies assess associations between patient and process factors and mortality, little research exists on how these factors are associated with non-mortality outcomes such as OFI. Understanding these factors can alert clinicians of patient groups prone to experiencing medical errors, and who are likely to benefit from quality improvement efforts, as an adjunct to existing trauma quality improvement programmes.

The aim of this study was to assess how patient and process factors are associated with OFI in trauma care.

## Methods

### Study design

We conducted a single-center registry-based retrospective cohort study. We used data from the Karolinska University Hospital trauma registry, which reports to the Swedish National Trauma Registry, [16] as well as the hospital’s local trauma care quality database. The content of the trauma registry has been previously described [17]. From 2017 and onwards the trauma care quality database includes all patients in the trauma registry as well as the results of the morbidity and mortality review, including identified OFI. We linked the trauma registry and trauma care quality database using personal identification numbers and extracted factors potentially associated with OFI.

### Setting

All major trauma patients in the greater metropolitan area of Stockholm are triaged to the Karolinska University Hospital in Solna, which is equivalent to a level one trauma center according to the criteria set by the American College of Surgeons [18]. The hospital has direct access to radiology, intervention, surgery, intensive care and consultants in all associated specialties [19, 20]. All trauma patients presenting to Karolinska University Hospital are included in a morbidity and mortality screening process that combines individual review by specialized nurses and audit filters, shown in Table 1. Patients identified as having a high potential for OFIs are discussed in multidisciplinary conferences. Examples of potential OFIs identified in this screening process include the need for more senior members assisting the trauma team or need for additional emergency operating rooms, which are then categorised into broader categories such as lack of resources and logistics. The multidisciplinary conferences take place every six to eight weeks and last about one hour. During the conferences, an average of ten patient cases are reviewed by experienced specialists from all the disciplines and professions involved in trauma care. The presence or absence of OFI is a consensus decision among all participants of the conference and is recorded in the trauma care quality database.

**Table 1:** Local audit filters

Audit filter	Number of patient cases flagged 2017–2021*
Systolic blood pressure under 90	87
Glasgow Coma Scale (GCS) less than 9 and not intubated	15
Injury Severity Score (ISS) more than 15 but not admitted to the ICU Injury Severity Score (ISS) more than 15 and no trauma team activation	516 259
No anticoagulation treatment within 72 h after traumatic brain injury time to acute intervention more than 60 min	211 823
Time to computed tomography (CT) more than 30 min	2444
Liver or spleen injury	213
Cardiopulmonary resuscitation with thoracotomy	32
Massive transfusion	151
Death within 30 days after trauma	203

*The total number of unique patients flagged was 3416. The sum of the number of patient cases flagged exceeds the number of unique patients flagged because several filters could flag the same patient

### Participants

The trauma registry includes all patients admitted with trauma team activation, regardless of Injury Severity Score (ISS), as well as patients admitted without trauma team activation but found to have an ISS of more than 9. We included all patients who had been included in the morbidity and mortality screening process between January 1, 2017 and June 1, 2021. We excluded patients who were younger than 15 years and patients who were dead on arrival.

### Variables

#### Study outcome

The outcome was the presence of OFI, as decided by the morbidity and mortality conference. An OFI is any failure of care including, but not limited to, any potentially preventable or preventable death, delay in treatment, clinical judgment error, missed diagnosis and technical error as decided by the mortality and morbidity conference. The study outcome is binary with the levels “Yes - At least one OFI identified” and “No - No OFI identified”.

#### Patient and process factors

We selected factors from the trauma registry, based on the locally used audit filters (Table 1), standard epidemiological factors and factors registered in the Swedish National Trauma Registry. The categorical factors were sex, survival after 30 days, highest hospital care level, Glasgow Coma Scale (GCS), respiratory rate, systolic blood pressure, working hours, weekend, time from arrival at the hospital until first computed tomography (CT) and if the patient was intubated. We also included the continuous factors age and ISS, and categorised these using standard cutoffs.

In the trauma registry, both systolic blood pressure and respiratory rate are registered as either a continuous value or a Revised Trauma Score category [21]. We converted the continuous values of systolic blood pressure and respiratory rate, if registered, and GCS score into the corresponding Revised Trauma Score category. If the patient was intubated and missing values for respiratory rate and GCS score, prehospital pre-intubation values were used.

The factor highest hospital care level, defined as the highest level of in-hospital care the patient being admitted to, is divided into five categories: emergency department, general ward, operating theatre, high dependency unit and critical care unit. The category emergency department means that the patient was not admitted, but was either discharged from or died in the emergency department. The category general ward is all wards with no further monitoring. The category operating theatre is assigned to patients who undergo surgery but who are not admitted to a high dependency or critical care unit post-operatively. High dependency units are wards with more extensive monitoring. Patients with multi-organ failure or who require mechanical ventilation are admitted to critical care units.

Factors denoting if the patient arrived at the hospital during working hours were included, defined as between 8.00 a.m. and 5 p.m., or during a weekend, defined as Saturday or Sunday.

### Statistical analysis

A complete case analysis was conducted after handling missing values in systolic blood pressure, respiratory rate, and GCS score as described above. We present sample characteristics using descriptive statistics. Bivariable logistic regression was used to determine unadjusted associations and multivariable logistic regression to determine adjusted associations between patient and process factors and OFI. We present odds ratios (OR) with associated 95% confidence intervals. A significance level of 5% was used. The programming language R was used for all analyses [22]. All statistical analysis was first done on synthetic data and later implemented on the data collected from the trauma registry and the trauma care quality database to ensure objectivity.

## Results

Out of 5,216 patients included in both the trauma registry and trauma care quality database between 2017 and 2021, 34 patients were excluded, leaving a total of 5,182 patients eligible for the study. 26 out of the 34 patients not eligible for the study because they were dead on arrival. Eight patients were excluded because they were aged less than 15 years or had missing data. The factor with the highest frequency of missing data was Glasgow Coma Scale, for which values were missing in 168 (3.2%) patients, nevertheless those patients were included.

Among the 5,182 patients included, there were 3,416 patients (65.9%) unique patients flagged with potential OFI, and 300 (5.8%) patients with OFI. Table 1 shows the number of patients flagged per audit filter. Sample characteristics are presented in Table 2. A total of 3,520 (68%) patients were male and 2,805 (54%) patients were adults in the age group 25–59 years. Most patients had ISS < 9, 2641 (51%). The number of patients who arrived outside of working hours was 3,060 (59.1%), and 1,671 (32.2%) patients arrived during weekends. A total of 203 (3.9%) patients died within 30 days.

**Table 2:** Sample characteristics

*Characteristic*	*No opportunity for improvement, N = 4,882* ^*1*^	*Opportunity for improvement, N = 300* ^*1*^	*Overall, N = 5,182* ^*1*^
***Sex***			
*Male*	*3,309 (68%)*	*211 (70%)*	*3,520 (68%)*
*Female*	*1,573 (32%)*	*89 (30%)*	*1,662 (32%)*
***Age (years)***			
*Older adolescents (15–19)*	*415 (8.5%)*	*15 (5.0%)*	*430 (8.3%)*
*Young adults (20–24)*	*500 (10%)*	*34 (11%)*	*534 (10%)*
*Adults (25–59)*	*2,670 (55%)*	*135 (45%)*	*2,805 (54%)*
*Older adults (60–100)*	*1,297 (27%)*	*116 (39%)*	*1,413 (27%)*
***Intubation***			
*None*	*4,459 (91%)*	*247 (82%)*	*4,706 (91%)*
*Pre-hospital*	*151 (3.1%)*	*12 (4.0%)*	*163 (3.1%)*
*Emergency department*	*272 (5.6%)*	*41 (14%)*	*313 (6.0%)*
***Highest hospital care level***			
*Emergency Department*	*1,116 (23%)*	*13 (4.3%)*	*1,129 (22%)*
*General Ward*	*1,980 (41%)*	*67 (22%)*	*2,047 (40%)*
*Operating Theater*	*867 (18%)*	*92 (31%)*	*959 (19%)*
*High Dependency Unit*	*184 (3.8%)*	*28 (9.3%)*	*212 (4.1%)*
*Critical Care Unit*	*735 (15%)*	*100 (33%)*	*835 (16%)*
***Respiratory rate***			
*10–29*	*4,504 (92%)*	*267 (89%)*	*4,771 (92%)*
*>29*	*152 (3.1%)*	*19 (6.3%)*	*171 (3.3%)*
*6–9*	*41 (0.8%)*	*0 (0%)*	*41 (0.8%)*
*1–5*	*15 (0.3%)*	*1 (0.3%)*	*16 (0.3%)*
*0*	*23 (0.5%)*	*0 (0%)*	*23 (0.4%)*
*Missing*	*147 (3.0%)*	*13 (4.3%)*	*160 (3.1%)*
***Systolic blood pressure***			
*>89*	*4,766 (98%)*	*284 (95%)*	*5,050 (97%)*
*76–89*	*63 (1.3%)*	*9 (3.0%)*	*72 (1.4%)*
*50–75*	*29 (0.6%)*	*7 (2.3%)*	*36 (0.7%)*
*1–49*	*1 (< 0.1%)*	*0 (0%)*	*1 (< 0.1%)*
*0*	*11 (0.2%)*	*0 (0%)*	*11 (0.2%)*
*Missing*	*12 (0.2%)*	*0 (0%)*	*12 (0.2%)*
***Glasgow Coma Scale***			
*13–15*	*4,304 (88%)*	*250 (83%)*	*4,554 (88%)*
*9–12*	*195 (4.0%)*	*20 (6.7%)*	*215 (4.1%)*
*3–8*	*226 (4.6%)*	*19 (6.3%)*	*245 (4.7%)*
*Missing*	*157 (3.2%)*	*11 (3.7%)*	*168 (3.2%)*
***Working hours*** ^***2***^			
*Yes*	*2,002 (41%)*	*120 (40%)*	*2,122 (41%)*
*No*	*2,880 (59%)*	*180 (60%)*	*3,060 (59%)*
***Weekend*** ^***3***^			
*Yes*	*1,577 (32%)*	*94 (31%)*	*1,671 (32%)*
*No*	*3,305 (68%)*	*206 (69%)*	*3,511 (68%)*
***Injury Severity Score***			
*Mild < 9*	*2,593 (53%)*	*48 (16%)*	*2,641 (51%)*
*Moderate 9–15*	*1,335 (27%)*	*82 (27%)*	*1,417 (27%)*
*Severe 16–25*	*545 (11%)*	*98 (33%)*	*643 (12%)*
*Profound > 25*	*409 (8.4%)*	*72 (24%)*	*481 (9.3%)*
***Time to first CT***			
*0–30*	*1,911 (39%)*	*95 (32%)*	*2,006 (39%)*
*No CT*	*510 (10%)*	*24 (8.0%)*	*534 (10%)*
*31–60*	*1,087 (22%)*	*85 (28%)*	*1,172 (23%)*
*61–90*	*524 (11%)*	*38 (13%)*	*562 (11%)*
*>90*	*850 (17%)*	*58 (19%)*	*908 (18%)*
***Survival after 30 days***			
*Alive*	*4,694 (96%)*	*285 (95%)*	*4,979 (96%)*
*Dead*	*188 (3.9%)*	*15 (5.0%)*	*203 (3.9%)*

^*1*^ *n (%)*

^*2*^ *Arrival at the hospital between 8.00 a.m. and 5 p.m*

^*3*^ *Arrival at the hospital on Saturday or Sunday*

The unadjusted and adjusted associations of selected factors with OFI are shown in Table 3.

**Table 3:** Unadjusted and adjusted logistic regression analyses of associations between patient level factor and opportunities for improvement

	*Unadjusted*			*Adjusted*		
*Characteristic*	*OR* ^*1*^	*95% CI* ^*1*^	*p-value*	*OR* ^*1*^	*95% CI* ^*1*^	*p-value*
***Sex***						
* Male*	*—*	*—*		*—*	*—*	
* Female*	*0.89*	*0.68, 1.14*	*0.36*	*1.04*	*0.79, 1.36*	*0.8*
***Age (years)***						
*Older adolescents (15–19)*	*—*	*—*		*—*	*—*	
*Young adults (20–24)*	*1.88*	*1.03, 3.60*	***0.046***	*1.87*	*0.98, 3.57*	*0.058*
*Adults (25–59)*	*1.40*	*0.84, 2.51*	*0.23*	*1.25*	*0.71, 2.20*	*0.4*
*Older adults (60–100)*	*2.47*	*1.48, 4.46*	***0.001***	*1.76*	*0.99, 3.14*	*0.055*
***Intubation***						
*None*	*—*	*—*		*—*	*—*	
*Pre-hospital*	*1.43*	*0.75, 2.51*	*0.24*	*0.93*	*0.41, 2.09*	*0.9*
*Emergency department*	*2.72*	*1.89, 3.83*	***< 0.001***	*1.07*	*0.66, 1.74*	*0.8*
***Highest hospital care level***						
*Emergency Department*	*—*	*—*		*—*	*—*	
*General Ward*	*2.90*	*1.65, 5.53*	***< 0.001***	*2.21*	*1.16, 4.20*	***0.016***
*Operating Theater*	*9.11*	*5.25, 17.2*	***< 0.001***	*4.80*	*2.46, 9.35*	***< 0.001***
*High Dependency Unit*	*13.1*	*6.78, 26.5*	***< 0.001***	*5.46*	*2.56, 11.7*	***< 0.001***
*Critical Care Unit*	*11.7*	*6.75, 22.0*	***< 0.001***	*4.67*	*2.23, 9.78*	***< 0.001***
***Respiratory rate***						
*10–29*	*—*	*—*		*—*	*—*	
*>29*	*2.11*	*1.25, 3.37*	***0.003***	*1.24*	*0.73, 2.10*	*0.4*
*6–9*^*2*^	*0.00*	*0.00, 17.9*	*0.97*	*-*	*-*	*-*
*1–5*	*1.12*	*0.06, 5.58*	*0.91*	*0.57*	*0.06, 5.21*	*0.6*
*0*^*2*^	*-*	*-*	*-*	*-*	*-*	*-*
*Missing*	*1.49*	*0.80, 2.56*	*0.18*	*1.00*	*0.40, 2.48*	*> 0.9*
***Systolic blood pressure***						
*>89*	*—*	*—*		*—*	*—*	
*76–89*	*2.40*	*1.10, 4.62*	***0.016***	*1.26*	*0.59, 2.68*	*0.5*
*50–75*	*4.05*	*1.62, 8.82*	***0.001***	*1.95*	*0.77, 4.95*	*0.2*
*1-49*^*2*^	*-*	*-*	*-*	*-*	*-*	*-*
*0*^*2*^	*-*	*-*	*-*	*-*	*-*	*-*
*Missing*^*2*^	*-*	*-*	*-*	*-*	*-*	*-*
***Glasgow Coma Scale***						
*13–15*	*—*	*—*		*—*	*—*	
*9–12*	*1.77*	*1.06, 2.78*	***0.020***	*0.92*	*0.54, 1.56*	*0.8*
*3–8*	*1.45*	*0.86, 2.29*	*0.14*	*0.76*	*0.39, 1.47*	*0.4*
*Missing*	*1.21*	*0.61, 2.15*	*0.56*	*0.39*	*0.15, 1.05*	*0.063*
***Working hours***						
*Yes*	*—*	*—*		*—*	*—*	
*No*	*1.04*	*0.82, 1.33*	*0.73*	*1.05*	*0.81, 1.35*	*0.7*
***Weekend***						
*Yes*	*—*	*—*		*—*	*—*	
*No*	*1.05*	*0.82, 1.35*	*0.73*	*1.07*	*0.82, 1.40*	*0.6*
***Injury Severity Score***						
*Mild < 9*	*—*	*—*		*—*	*—*	
*Moderate 9–15*	*3.32*	*2.32, 4.80*	***< 0.001***	*2.03*	*1.36, 3.03*	***< 0.001***
*Severe 16–25*	*9.71*	*6.83, 14.0*	***< 0.001***	*5.15*	*3.39, 7.82*	***< 0.001***
*Profound > 25*	*9.51*	*6.53, 14.0*	***< 0.001***	*5.54*	*3.43, 8.96*	***< 0.001***
***Time to first CT***						
*0–30*	*—*	*—*		*—*	*—*	
*No CT*	*0.95*	*0.59, 1.47*	*0.81*	*0.81*	*0.50, 1.32*	*0.4*
*31–60*	*1.57*	*1.16, 2.13*	***0.003***	*1.53*	*1.11, 2.10*	***0.009***
*61–90*	*1.46*	*0.98, 2.13*	*0.057*	*2.44*	*1.60, 3.72*	***< 0.001***
*>90*	*1.37*	*0.98, 1.92*	*0.065*	*1.60*	*1.11, 2.31*	***0.012***
***Survival after 30 days***						
*Alive*	*—*	*—*		*—*	*—*	
*Dead*	*1.31*	*0.74, 2.18*	*0.32*	*0.66*	*0.36, 1.20*	*0.2*

^*1*^ *OR = Odds Ratio, CI = Confidence Interval*

^*2*^ *Not enough observations in this group to allow one or several of its coefficients and CIs to be estimated*

The following factors were significantly associated with OFI only in the unadjusted analysis: Young adults (aged 20–24 years) (OR 1.88, 95% CI 1.03–3.60, p-value 0.046) and older adults (aged 60–100 years) (OR 2.47, 95% CI 1.48–4.46, p-value 0.001) compared with older adolescents (aged 15–19 years). Intubation in the emergency department compared with no intubation (OR 2.72, 95% CI 1.89–3.83, p-value < 0.001). Respiratory rate higher than 29 compared with respiratory rate between 10 and 29 (OR 2.11, 95% CI 1.25–3.37, p-value 0.003). Systolic blood pressure between 50 and 75 (OR 4.05, 95% CI 1.62–8.82, p-value 0.001) and between 76 and 89 (OR 2.40, 95% CI 1.10–4.62, p-value 0.016) compared with a systolic blood pressure of more than 89. Glasgow coma scale 9–12 (OR 1.77, 95% CI 1.06–2.78, p-value 0.020) compared with Glasgow coma scale 13–15.

Compared to care in the emergency department, all higher levels of care were significantly associated with increased odds of OFI in both the unadjusted and adjusted analyses. The highest odds were found in patients treated in a high dependency unit (aOR 8.25, 95% CI 4.02–16.9, p-value < 0.001). Compared to mild trauma (ISS < 9), all other ISS groups had significantly higher odds of OFI, with the highest odds of OFI in those with profound trauma (ISS > 25) (aOR 5.54, 95% CI 3.43–8.96, p-value < 0.001).

A time to first CT between 31 and 60 min was in the unadjusted analysis significantly associated with higher odds of OFI compared with a time to first CT between 0 and 30 min (OR 1.57, 95% CI 1.16–2.13, p-value 0.003). This association remained significant in the adjusted analysis (OR 1.53, 95% CI 1.11–2.10, p-value 0.009), but in this analysis a time to first CT between 61 and 90 min (aOR 2.44, 95% CI 1.60–3.72, p-value < 0.001) and a time over 90 min (aOR 1.60, 95% CI 1.11–2.31, p-value 0.012) were also associated with significantly higher odds of OFI.

Sex, arrival on weekday or weekend and arrival during working hours and survival after 30 days were not significantly associated with OFI in neither the unadjusted nor the adjusted analysis.

## Discussion

We found that higher levels of care were associated with increased odds of OFI. Previous research indicates that more than half of the preventable and probably preventable trauma deaths occur in critical care units, [13] and that the most common OFI were related to airway management and perioperative care 23. These findings could be because patients admitted to higher levels of care receive more interventions, increasing the probability of OFI. Similarly, we found ISS to be significantly associated with OFI. This could be because the management of severely injured patients is more complex, and they require more interventions, and that their care is therefore more prone to OFI. Interestingly, the vital signs systolic blood pressure, respiratory rate, and GCS as well as intubation were all significantly associated with OFI in the unadjusted analysis but not in the adjusted analysis. This could be because these factors indicate more severe trauma, which was more effectively captured by ISS in the adjusted analysis.

We also found that time to CT was associated with increased odds of OFI. Delays to CT in trauma patients has in previous studies been associated with poor outcomes, [24] and a study recently made in Japan found that CT within ten minutes after arrival in severe trauma patients was associated with decreased in-hospital mortality [25]. The association between time to CT and OFI is complex because even though life saving interventions need to be prioritised over imaging, this can result in missed injuries for which management is then delayed, potentially resulting in complications. Our finding that no CT was not significantly associated with OFI compared to a time to CT between 0 and 30 min may be because these patients are either so mildly injured that they can be discharged from the emergency department, or that they are so severely injured that they do not survive until CT.

Our study had several limitations. Although all trauma patients were reviewed by specialized nurses, the selection for review by the morbidity and mortality conference relied mostly on audit filters, meaning that there is a risk for misclassification whereby some patients with OFI were not selected for review by the conference and therefore registered as patients without OFI. Another limitation is that several potential relevant clinical factors, such as mental status or airway difficulty, were not available in the registry and therefore not adjusted for in the analysis. Furthermore, this study was a single-center study, and its results illustrate the situation in Stockholm.

## Conclusion

The care of patients who require higher levels of care, have more severe trauma, and who’s CT is delayed, has significantly higher odds of OFI. These factors represent reasonable targets for new initiatives to supplement ongoing trauma quality improvement efforts.

## Data Availability

The data that support the findings of this study are available following the approval of a project suggesting using the data by the Swedish Ethical Review Authority and the appropriate bodies at the Karolinska University Hospital. More information is available on request from the corresponding author, H. Albaaj. The data are not publicly available due to containing information that could compromise the privacy of research participants.

## References

[1] Daniels David S, Roy N, Solomon H, Stålsby Lundborg C, Gerdin Wärnberg M. Measuring post-discharge socioeconomic and quality of life outcomes in trauma patients: A scoping review. *Journal of Patient-Reported Outcomes* [Internet]. Springer Science; Business Media LLC; 2021; 5. 10.1186/s41687-021-00346-6. 10.1186/s41687-021-00346-6 PMC8353045 34370128

[2] Corso P, Finkelstein E, Miller T, Fiebelkorn I, Zaloshnja E. Incidence and lifetime costs of injuries in the United States. *Injury Prevention* [Internet]. BMJ; 2015; 21: 434–440. 10.1136/ip.2005.010983rep. 10.1136/ip.2005.010983rep 26609059

[3] James SL, Castle CD, Dingels ZV, Fox JT, Hamilton EB, Liu Z et al. Estimating global injuries morbidity and mortality: methods and data used in the Global Burden of Disease 2017 study. *Injury Prevention* [Internet]. BMJ; 2020; 26: i125–i153. 10.1136/injuryprev-2019-043531. 10.1136/injuryprev-2019-043531 PMC7571362 32839249

[4] Murray CJ, Lopez AD, Organization WH, et al. The global burden of Disease: a comprehensive assessment of mortality and disability from Diseases, injuries, and risk factors in 1990 and projected to 2020: summary. World Health Organization; 1996.

[5] Haagsma JA et al. The global burden of injury: incidence, mortality, disability-adjusted life years and time trends from the Global Burden of Disease study 2013. *Injury Prevention* [Internet]. BMJ; 2015; 22: 3–18. 10.1136/injuryprev-2015-041616. 10.1136/injuryprev-2015-041616 PMC4752630 26635210

[6] Prin M, Li G. Complications and in-hospital mortality in trauma patients treated in intensive care units in the United States, 2013. *Injury Epidemiology* [Internet]. Springer Science; Business Media LLC; 2016; 3. 10.1186/s40621-016-0084-5. 10.1186/s40621-016-0084-5 PMC4974260 27747555

[7] Donabedian A, American Medical Association (AMA). The Quality of Care. *JAMA* [Internet]. ; 1988; 260: 1743. 10.1001/jama.1988.03410120089033.

[8] World Health Organization., Guidelines for trauma quality improvement programmes. World Health Organization [Internet]. [cited 2022]. Available from: https://apps.who.int/iris/bitstream/handle/10665/44061/9789241597746%7B/_%7Deng.pdf;jsessionid=E90A5736D4F3786CCBAA19E19E4EEF5F?sequence=1.

[9] Ghorbani P, Strömmer L. Analysis of preventable deaths and errors in trauma care in a Scandinavian trauma level-I centre. *Acta Anaesthesiologica Scandinavica* [Internet]. Wiley; 2018; 62: 1146–1153. 10.1111/aas.13151. 10.1111/aas.13151 29797712

[10] Esposito TJ, Sanddal TL, Reynolds SA, Sanddal ND. Effect of a Voluntary Trauma System on Preventable Death and Inappropriate Care in a Rural State. *The Journal of Trauma: Injury, Infection, and Critical Care* [Internet]. Ovid Technologies (Wolters Kluwer Health); 2003; 54: 663–670. https://doi.org/10.1097%2F01.ta.0000058124.78958.6b. 10.1097/01.TA.0000058124.78958.6B 12707527

[11] Sanddal TL, Esposito TJ, Whitney JR, Hartford D, Taillac PP, Mann NC et al. Analysis of Preventable Trauma Deaths and Opportunities for Trauma Care Improvement in Utah. *Journal of Trauma: Injury, Infection and Critical Care* [Internet]. Ovid Technologies (Wolters Kluwer Health); 2011; 70: 970–977. https://doi.org/10.1097%2Fta.0b013e3181fec9ba. 10.1097/TA.0b013e3181fec9ba 21206286

[12] Zafarghandi M-R, Saeed Modaghegh M-H, Sayyar Roudsari B. Preventable Trauma Death in Tehran: An Estimate of Trauma Care Quality in Teaching Hospitals. *The Journal of Trauma: Injury, Infection, and Critical Care* [Internet]. Ovid Technologies (Wolters Kluwer Health); 2003; 55: 459–465. 10.1097/01.ta.0000027132.39340.fe. 10.1097/01.TA.0000027132.39340.FE 14501887

[13] Teixeira PGR, Inaba K, Hadjizacharia P, Brown C, Salim A, Rhee P et al. Preventable or Potentially Preventable Mortality at a Mature Trauma Center. *Journal of Trauma: Injury, Infection, and Critical Care* [Internet]. Ovid Technologies (Wolters Kluwer Health); 2007; 63: 1338–1347. 10.1097/ta.0b013e31815078ae. 10.1097/TA.0b013e31815078ae 18212658

[14] O’Reilly D, Mahendran K, West A, Shirley P, Walsh M, Tai N. Opportunities for improvement in the management of patients who die from haemorrhage after trauma. *British Journal of Surgery* [Internet]. Oxford University Press (OUP); 2013; 100: 749–755. 10.1002/bjs.9096. 10.1002/bjs.9096 23483534

[15] Roy N, Kizhakke Veetil D, Khajanchi MU, Kumar V, Solomon H, Kamble J et al. Learning from 2523 trauma deaths in india- opportunities to prevent in-hospital deaths. *BMC Health Services Research* [Internet]. Springer Science; Business Media LLC; 2017; 17. https://doi.org/10.1186%2Fs12913-017-2085-7. 10.1186/s12913-017-2085-7 PMC5314603 28209192

[16] Swetrau ÅrsrapportST. 2019. Annual report, Stockholm: Svenska Traumaregister [Internet]. [cited 2022]. Available from: https://rcsyd.se/swetrau/wp-content/uploads/sites/10/2020/09/A%CC%8Arsrapport-SweTrau-2019.pdf.

[17] Ringdal KG, Coats TJ, Lefering R, Bartolomeo SD, Steen PA, Roise O et al. The utstein template for uniform reporting of data following major trauma: A joint revision by SCANTEM, TARN, DGU-TR and RITG. *Scandinavian Journal of Trauma, Resuscitation and Emergency Medicine* [Internet]. Springer Science; Business Media LLC; 2008; 16: 7. 10.1186/1757-7241-16-7. 10.1186/1757-7241-16-7 PMC2568949 18957069

[18] American College of Surgeons Committee on Trauma (2015). Statement on trauma center designation based upon system need. Bull Am Coll Surg.

[19] Socialstyrelsen, Traumavård vid allvarlig händelse. Socialstyrelsen 2015 [Internet]. [cited 2022]. Available from: https://www.socialstyrelsen.se/globalassets/sharepoint-dokument/artikelkatalog/ovrigt/2015-11-5.pdf.

[20] Karolinska Universitetssjukhuset Solna., Traumamanual Karolinska Universitetssjukhuset Solna 2020 [Internet]. [cited 2022]. Available from: https://traumarummet.files.wordpress.com/2020/09/traumamanualen-2020.pdf.

[21] Champion HR, Sacco WJ, Copes WS, Gann DS, Gennarelli TA, Flanagan ME. A Revision of the Trauma Score. *The Journal of Trauma: Injury, Infection, and Critical Care* [Internet]. Ovid Technologies (Wolters Kluwer Health); 1989; 29: 623–629. 10.1097/00005373-198905000-00017. 10.1097/00005373-198905000-00017 2657085

[22] RStudio Team. RStudio: Integrated Development Environment for R [Internet]. Boston, MA: RStudio, PBC. ; 2020. Available from: http://www.rstudio.com/.

[23] Teixeira PGR. Preventable Morbidity at a Mature Trauma Center. *Archives of Surgery* [Internet]. American Medical Association (AMA); 2009; 144: 536. 10.1001/archsurg.2009.82. 10.1001/archsurg.2009.82 19528387

[24] Katayama Y, Kitamura T, Hirose T, Kiguchi T, Matsuyama T, Sado J et al. Delay of computed tomography is associated with poor outcome in patients with blunt traumatic aortic injury. *Medicine* [Internet]. Ovid Technologies (Wolters Kluwer Health); 2018; 97: e12112. 10.1097/md.0000000000012112. 10.1097/MD.0000000000012112 PMC6392548 30170440

[25] Yamamoto R, Suzuki M, Funabiki T, Sasaki J. Immediate CT after hospital arrival and decreased in-hospital mortality in severely injured trauma patients. *BJS Open* [Internet]. Oxford University Press (OUP); 2023; 7. 10.1093/bjsopen/zrac133. 10.1093/bjsopen/zrac133 PMC9866241 36680778

